# Investigating Skin Cancer Risk and Sun Safety Practices Among LGBTQ+ Communities in Canada

**DOI:** 10.3390/curroncol31120593

**Published:** 2024-12-19

**Authors:** François Lagacé, Farhan Mahmood, Santina Conte, Lorena A. Mija, Amina Moustaqim-Barrette, Jonathan LeBeau, Alyson McKenna, Mahan Maazi, Johnny Hanna, Alexandra Sarah Victoria Kelly, Raquel Lazarowitz, Elham Rahme, Travis J. Hrubeniuk, Ellen Sweeney, Ivan V. Litvinov

**Affiliations:** 1 Division of Dermatology, McGill University, Montréal, QC H4A 3J1, Canada; francois.lagace@mail.mcgill.ca (F.L.); farhan.mahmood@mail.mcgill.ca (F.M.); santina.conte@mail.mcgill.ca (S.C.); 2 Faculté de Médecine, Université de Montréal, Montréal, QC H3T 1J4, Canada; 3 Faculty of Medicine, McGill University, Montréal, QC H4A 3J1, Canada; amina.moustaqim-barrette@mail.mcgill.ca (A.M.-B.); raquel.lazarowitz@mail.mcgill.ca (R.L.); 4 Faculty of Medicine, University of British Columbia, Vancouver, BC V6T 1Z3, Canada; 5 Faculté de Médecine, Université Laval, Québec, QC G1V 0A6, Canada; 6 Faculty of Sciences, University of Ottawa, Ottawa, ON K1N 9A4, Canada; 7 Division of Clinical Epidemiology, McGill University, Montréal, QC H4A 3J1, Canada; elham.rahme@mcgill.ca; 8 Manitoba Tomorrow Project, Cancer Care Manitoba, Winnipeg, MB R3E 0W2, Canada; thrubeniuk@cancercare.mb.ca; 9 Community Health Sciences, Max Rady College of Medicine, University of Manitoba, Winnipeg, MB R3E 0W2, Canada; 10 Atlantic PATH, Faculty of Medicine, Dalhousie University, Halifax, NS B3H 4R2, Canada; ellen.sweeney@dal.ca

**Keywords:** sun protection, skin cancer risk, LGBTQ+ health, sun safety, Canadian health behavior

## Abstract

Background: Skin cancer prevention relies on effective sun safety practices. Previous studies have shown that LGBTQ+ individuals exhibit lower sunscreen use and higher tanning bed usage compared to their non-LGBTQ+ counterparts. This study is the first to assess skin cancer risk factors, sun-protective behaviors, and skin cancer concerns among LGBTQ+ individuals across Canada. Methods: A national survey study was conducted between July 2020 and March 2024 and included LGBTQ+ respondents aged ≥ 16 years who had completed the survey. Responses were summarized using frequency counts/percentages for categorical variables and means/standard deviations for continuous variables. Logistic regression models were used to calculate age- and gender-adjusted odds ratios for subgroup analyses. Results: Of the 700 LGBTQ+ participants included (59.3% women; median age 38 years), the majority had a Fitzpatrick skin phototype (FSP) I–III (76.4%). Concerningly, 60% reported >10 lifetime sunburns, 58% reported ≥1 blistering sunburn, 34% had used a tanning bed ≥1 time in their lifetime, and 69% reported having a tan in the last 12 months. Sunscreen was worn regularly by only half of the respondents, and half of the participants agreed or strongly agreed with “I look better and/or healthier with a tan”. Additional comparisons are presented based on gender, FSP, education, and income. Conclusion: The findings of this study highlight the need for public health campaigns tailored to the LGBTQ+ community, emphasizing culturally sensitive sun safety education, particularly for LGBTQ+ men, individuals with FSP IV–VI, and those with lower education levels, to help reduce future skin cancer risk.

## 1. Introduction

Cutaneous melanoma (CM) is a skin cancer associated with the highest burden of morbidity, mortality, and years of potential life lost [1], and its incidence is rapidly increasing worldwide [2,3]. Our research group previously found that the overall incidence rate of CM in Canada increased from 12.29 per 100,000 individuals per year during 1992–2010 to 20.75 during 2011–2017 [4,5]. Many behavioral, geographic, and demographic risk factors contribute to CM risk; however, one of the most important modifiable risk factors is ultraviolet (UV) exposure [6,7,8].

Our team has previously examined ultraviolet (UV) exposure, sun-protective behaviors, levels of worry, and baseline CM knowledge in Atlantic Canada [9]. Upon stratifying the data by sexual orientation, there were no significant differences in lifetime sunburns, tanning bed use, or total sun exposure between self-identified LGBTQ+ and non-LGBTQ+ populations. However, differences between the two groups were noted with regard to sunscreen usage, whereby LGBTQ+ respondents were less likely to use sunscreen compared to their non-LGBTQ+ counterparts. On the other hand, they were more likely to use sunscreen with a sun protection factor (SPF) ≥ 30, wear hats, and reported less frequent tans in the past 12 months [9]. Differences in sun safety behaviors between heterosexual and LGBTQ+ communities have also been noted in studies conducted across the United States (US), with gay and bisexual men using tanning devices or sunless tanning more frequently than heterosexual men [10,11,12,13] and heterosexual women engaging in indoor tanning more than lesbian and bisexual women [13]. Finally, American sexual minority men (gay or bisexual) were found to use less sun-protective clothing but more sunscreen compared to their heterosexual counterparts [12].

Some studies report a higher prevalence of skin cancer among sexual minority males compared to heterosexual males [13,14], while the data is less consistent in their female counterparts [14,15]. Moreover, gender non-conforming individuals have been reported to have a higher lifetime prevalence of skin cancers [16].

The increased prevalence of skin cancer among sexual minority men and gender-diverse individuals is an important reason to assess skin cancer risk factors and sun protection habits among sexual minorities in Canada. An acknowledgment of such differences can help guide physicians’ counseling efforts and allow for the development of culturally competent public health interventions, specifically focusing on the needs of the LGBTQ+ population that already faces important health disparities [17]. This study serves as the first large-scale assessment of UV exposure, sun-protective behaviors, and levels of worry for CM among LGBTQ+ individuals across Canada, expanding on our previous study [9]. It also compares these variables between genders, education levels, income levels, and Fitzpatrick skin phototype (FSP) within the LGBTQ+ community.

## 2. Methods

We used the Checklist for Reporting Results of Internet E-Surveys (CHERRIES) to report our study design [18].

### 2.1. Study Design

A survey-based study was conducted across the Canadian population. Given that approximately 4% of the Canadian population identifies as LGBTQ+ [19], a minimum sample size calculation using a 95% confidence interval (CI) with a 5% margin of error determined that 385 participants was sufficient. Our final sample consisted of 700 participants who identified as LGBTQ+, surpassing the minimum requirement and enabling us to perform more detailed subgroup analyses.

### 2.2. Ethics Statement

This study (study number A04-B16-20B) was granted approval by the Research Ethics Board of McGill University. Prior to survey completion, all participants provided informed consent electronically. All participants were given comprehensive descriptions regarding the survey’s duration, data storage procedures, research team composition, and study objectives. Data was securely stored on a protected network, accessible only to authorized research team members through a two-factor authentication, in compliance with the principles outlined in the General Data Protection Regulations.

### 2.3. Development and Pre-Testing

Participants completed an electronically administered, validated, and reliable patient questionnaire: the Sun Exposure and Behavior Inventory (SEBI) [20]. This survey was modified to meet our study’s needs and encompassed a range of outcomes, including demographic factors such as participant sexuality and gender, UV exposure, sun-protective behaviors, knowledge pertaining to CM, and the degree of concern regarding CM. In keeping with our prior publications [9,21], which utilized this identical survey instrument, we collaborated with the Maelstrom Research [22] team to ensure that questions were clear, direct, and objective. To maintain data integrity, the survey employed adaptive questioning techniques, permitting respondents to select options such as “not applicable” or “I would rather not say”. Participants were also able to modify their answers prior to submission.

### 2.4. Recruitment Process and Survey Administration

Survey responses were collected between July 2020 and March 2024. Participants voluntarily completed the survey electronically through one of two online platforms (https://sunfit.mytrial.me/survey, our new survey platform developed by our team in June 2023, or https://bit.ly/3TxKnl1, developed by our team in June 2020). Recruitment strategies included social media advertisements, in-person events, and newsletters. Additional recruitment methods included emails that were sent to participants who had previously completed the survey and consented to further communication. These emails encouraged participants to invite friends and family to complete the online survey. Some of the participants were recruited with the help of longitudinal cohorts, notably the Manitoba Tomorrow Project and Atlantic PATH, regional cohorts of the Canadian Partnership for Tomorrow’s Health (CanPath) [23,24], as previously reported [9,21]. Given the open nature of some of the recruitment strategies, an overall response rate could not be calculated.

All participants identifying as LGBTQ+ aged ≥16 years with a complete survey were eligible. As part of the survey, all respondents were asked the following question: do you identify with the LGBTQ+ community (lesbian, gay, bisexual, transgender, queer, other)? And were able to respond ‘Yes’, ‘No’, ‘Would rather not say’, or ‘Do not know’. All participants who indicated ‘Yes’ to this question were considered to identify as LGBTQ+ for the purpose of this analysis. Participants living outside Canada who did not identify as LGBTQ+, who were younger than 16 years, or who had incomplete surveys were excluded. In total, 734 participants who identified with the LGBTQ+ community submitted a complete survey. Of these, 5 were excluded due to their age, and 11 were excluded on the basis of non-Canadian residence. Based on email addresses, we identified 18 duplicate entries. Only the most recent entry was kept, resulting in a final cohort of 700 participants. This investigation evaluated 23 out of 42 questionnaire items, covering demographic inquiries, UV exposure and skin cancer history, sun protection practices, and level of concern.

### 2.5. Statistical Analysis

Baseline demographic characteristics and survey responses were summarized using frequency counts and percentages for categorical variables and mean with standard deviations for continuous variables. Participants were stratified based on gender identity (men vs. women vs. gender diverse), FSP (types I–III vs. types IV–VI), level of education (university degree or higher vs. no university degree), and household annual income (≥CAD 50,000 vs. <CAD 50,000). Those with FSP IV–VI were considered to have a skin of color, as previously described [25]. Logistic regression models were used to determine age- and gender-adjusted odds ratios (aORs) and their corresponding 95% CI that were used to compare outcomes of interest between groups. *p*-values < 0.05 were considered statistically significant.

## 3. Results

A total of 700 participants who identified as LGBTQ+ completed the survey. Women represented 59.3% of the respondents with a mean age of 40.4 years (standard deviation 17.1 years). The majority (84.7%) of respondents identified as non-Hispanic White or European Canadian, many of whom resided in Quebec (20.0%) and Manitoba (19.9%). Table 1 provides further details on the demographic data of respondents. As per the Manitoba Tomorrow Project, all frequency counts less than 10 must be reported as <10 to ensure patient confidentiality.

In total, 5.7% of participants reported a personal history of skin cancer, including 14 cases of CM. Concerningly, 60.0% have had ≥10 lifetime sunburns, 58.2% reported ≥1 blistering sunburn, 34.3% of participants had used a tanning bed at least once in their lifetime, and 68.9% reported having a tan in the last 12 months. The sun protection method that was the most frequently regularly practiced was the use of long-sleeved shirts (64.6%), while the least practiced were hat-wearing (25.3%) and shade-seeking (32.4%). In terms of sunscreen, 63.4% used broad spectrum (UVA and UVB), and 86.3% used SPF 30+. Conversely, 6.3% do not wear sunscreen, and 29.4% do not know if their sunscreen offers broad-spectrum coverage. Most participants check their skin for new or changing moles (73.7%), and most would be worried if a mole became irregular in shape (95.7%), changed in color (97.3%), or grew in size (96.3%). Notably, 50.0% of participants agreed or strongly agreed with the statement “I look better and/or healthier with a tan”, 34.6% with “sunscreens pollute the oceans”, and 15.9% with “having a base tan is protective against the sun’s UV radiation/skin damage”. The majority (82.7%) disagreed or strongly disagreed with “tanning beds are a safer, more controlled way to get a tan than from the sun”. Additional details on the respondents’ UV exposure, melanoma risk factors, tanning and sun protective habits and knowledge, and level of worry for CM are presented in Supplementary Tables S1 and S2.

### 3.1. Gender Identity

**Figure 1:**
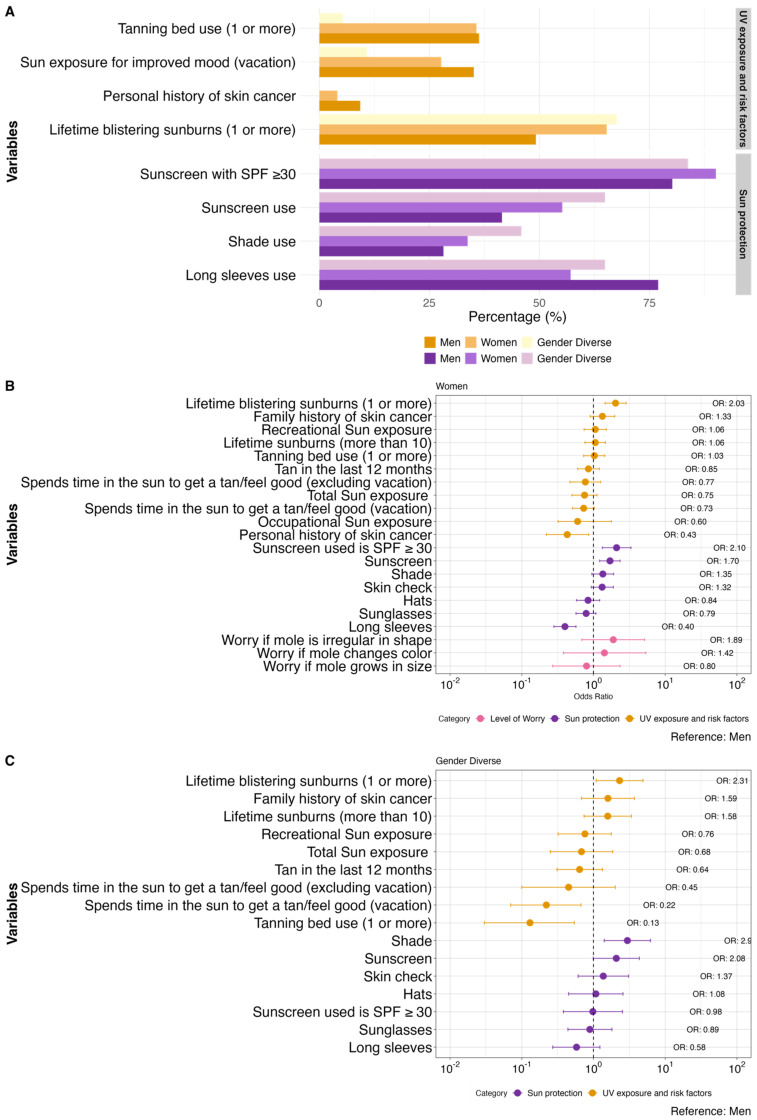
Comparison of sun exposure, CM risk factors, sun protection habits, and level of worry for CM between women (n = 415), men (n = 248), and gender-diverse individuals (n = 37). (**A**) Bar graph depicting the statistically significant variables. (**B**) Forest plot with all the variables comparing women to men. (**C**) Forest plot with all the variables comparing gender diverse individuals to men. Odds ratios (OR) are adjusted for age and gender where appropriate.

Compared to LGBTQ+ men (n = 248), LGBTQ+ women (n = 415) reported a significantly lower personal history of skin cancer (aOR 0.43, 0.22–0.86) and significantly more lifetime blistering sunburns (aOR 2.03, 1.45–2.85). In terms of photoprotection, women used more sunscreen (aOR 1.70, 1.22–2.35) and SPF 30+ (aOR 2.10, 1.33–3.33), but less long-sleeved shirts (aOR 0.40, 0.28–0.57). Interestingly, there were no statistically significant differences between LGBTQ+ men and women in terms of tanning bed use, total/recreational/occupation sun exposure, tanning in the last 12 months, or time spent in the sun daily or multiple days per week to get a tan or to feel good. Gender-diverse participants (n = 37) were significantly less likely to report using tanning beds compared to LGBTQ+ women (aOR 0.13, 0.03–0.54) and LGBTQ+ men (aOR 0.13, 0.03–0.54) and were less likely to spend time in the sun for tanning or leisure purposes while on vacation when compared to both women (aOR 0.31, 0.11–0.92) and men (aOR 0.22, 0.07–0.67). Blistering sunburns were more common in LGBTQ+ men compared to gender-diverse individuals (aOR 2.31, 1.09–4.90). Regarding sun protection, shade-seeking was more common in men than gender-diverse individuals (aOR 2.97, 1.41–6.24). Additional details are presented in Figure 1 and Supplementary Table S3.

### 3.2. Fitzpatrick Skin Phototype

**Figure 2:**
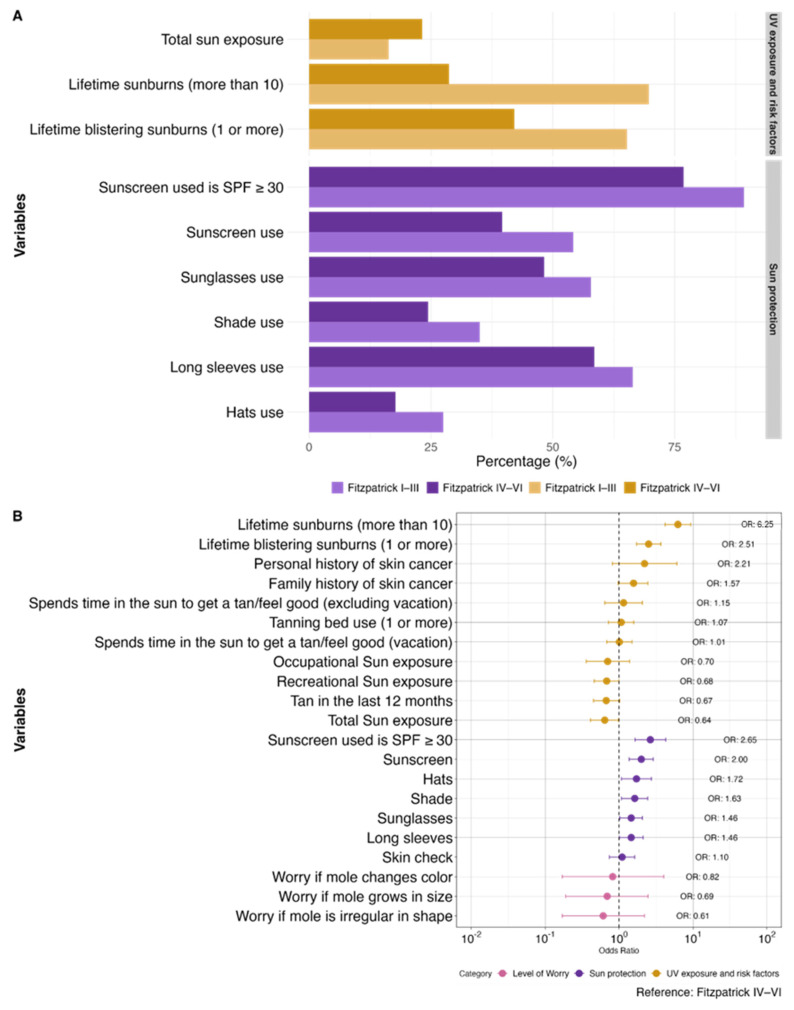
Comparison of sun exposure, CM risk factors, sun protection habits, and level of worry for CM between Fitzpatrick skin phototypes I–III (n = 535) vs. phototypes IV–VI (n = 164) as a bar graph with the statistically significant variables (**A**) and corresponding forest plot with all variables (**B**). Odds ratios (OR) are adjusted for age and gender where appropriate.

When comparing LGBTQ+ respondents of different FSPs [26], respondents with FSP I–III (n = 535) were significantly more likely to report lifetime sunburns (aOR 6.25, 4.20–9.30) and blistering sunburns (aOR 2.51, 1.72–3.67). Individuals with FSP I–III also used more sun protection measures across all variables, namely sunscreen (aOR 2.00, 1.37–2.90), long sleeves (aOR 1.46, 1.01–2.11), hats (aOR 1.72, 1.08–2.74), sunglasses (aOR 1.46, 1.02–2.07), SPF ≥ 30 (aOR 2.65, 1.65–4.27), and shade seeking (aOR 1.63, 1.08–2.44), compared to respondents with FSP IV–VI (n = 164). In contrast, respondents with FSP I–III reported less total sun exposure (aOR 0.64, 0.41–0.98) than those with FSP IV–VI. The majority (>95%) in both groups would worry if a mole was irregularly shaped or changed in color or size. Further details are presented in Figure 2 and Supplementary Table S4.

### 3.3. Education

**Figure 3:**
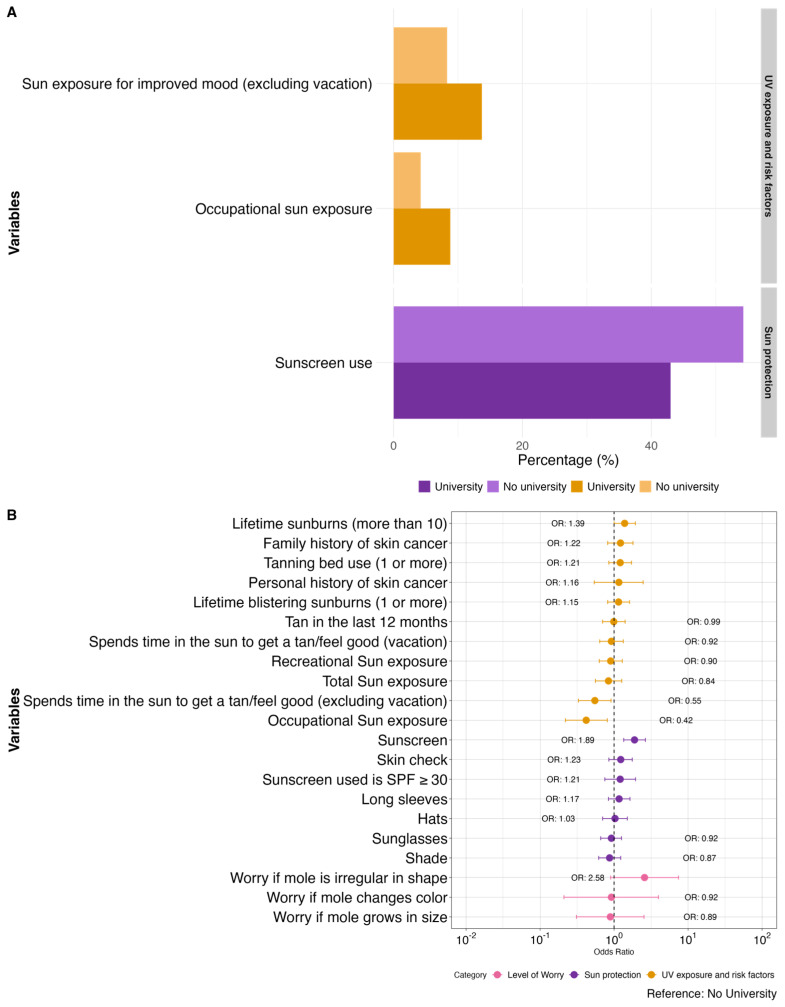
Comparison of sun exposure, CM risk factors, sun protection habits, and level of worry for CM between those who have completed a university degree (n = 409) vs. those who have not completed a university degree (n = 249), depicted as a bar graph with the statistically significant variables (**A**) and corresponding forest plot with all variables (**B**). Odds ratios (OR) are adjusted for age and gender where appropriate.

Respondents with a university degree (n = 409) reported significantly less occupational sun exposure (aOR 0.42, 0.22–0.81) and spent less time in the sun with the intention of feeling good or getting a tan (aOR 0.55, 0.33–0.91) while using more sunscreen (aOR 1.89, 1.35–2.65) when compared to respondents without a university degree (n = 249). Additional details are provided in Figure 3 and Supplementary Table S5.

### 3.4. Income

**Figure 4:**
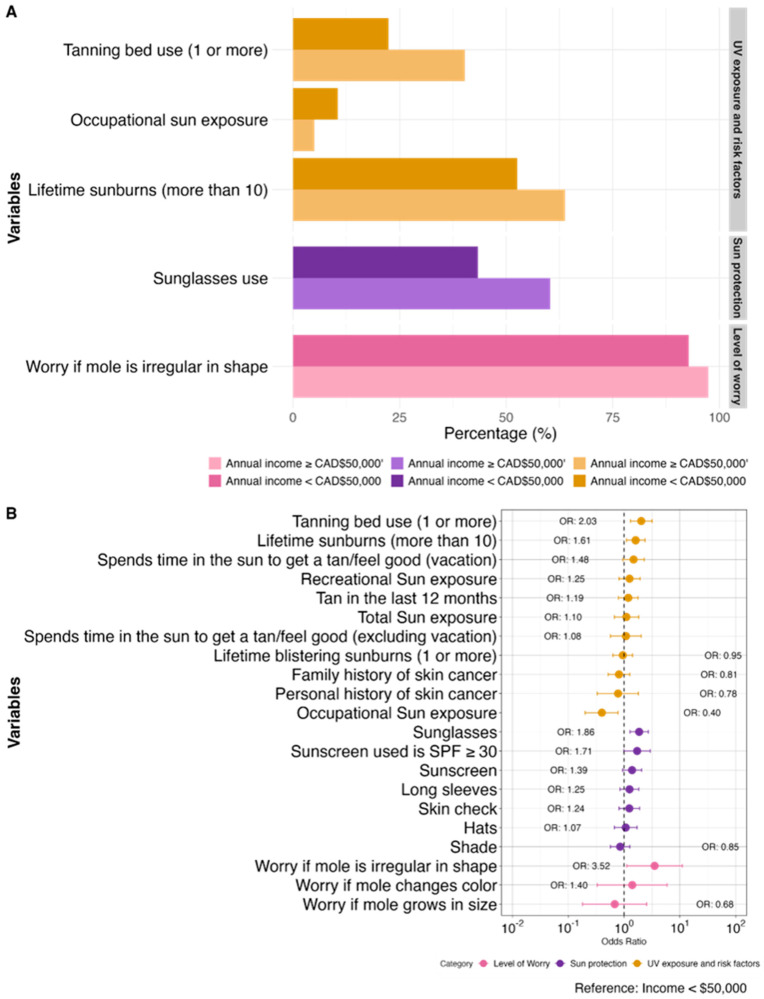
Comparison of sun exposure, CM risk factors, sun protection habits, and level of worry for CM between individuals with an annual household income ≥ CAD 50,000 (n = 459) vs. individuals with an annual income < CAD 50,000 (n = 152) as a bar graph with the statistically significant variables (**A**) and corresponding forest plot with all variables (**B**). Odds ratios (OR) are adjusted for age and gender where appropriate.

Those with an annual household income ≥ CAD 50,000 after taxes (n = 459) had significantly more lifetime sunburns (aOR 1.61, 1.10–2.38) and tanning bed usage (aOR 2.03, 1.30–3.17), but less occupational sun exposure (aOR 0.40, 0.20–0.78), compared to those with an annual household income < CAD 50,000 (n = 152). The only significant difference in sun protective practices was with regards to the wearing of sunglasses, which was more frequently practiced by those with an income ≥ CAD 50,000 (aOR 1.86, 1.28–2.72). Additionally, those with a higher income were more likely to be worried if a mole became irregular in shape (aOR 3.52, 1.12–11.07). Additional details are presented in Figure 4 and Supplementary Table S6.

## 4. Discussion

Our study is the first comprehensive analysis, to our knowledge, examining skin cancer risk factors, sun protection behaviors, and skin cancer concerns among the Canadian LGBTQ+ population. We compared our findings with two previous studies conducted by our team in Manitoba and the Atlantic provinces of Nova Scotia, New Brunswick, Prince Edward Island, and Newfoundland and Labrador (Supplementary Table S7). Since LGBTQ+ respondents in the prior studies comprised only 2.9–4.2% of the sample, the results primarily reflect non-LGBTQ+ individuals.

In this current study, 5.7% of respondents reported a personal history of skin cancer, which was more common among LGBTQ+ men compared to women. Other studies have also revealed differing rates of skin cancer among members of the LGBTQ+ community. When compared to cisgender men, transgender men and transgender women did not have an increased risk of skin cancer, whereas gender non-conforming individuals did [16]. Data from the National Health Interview Survey in the United States revealed a higher prevalence of CM among lesbian and bisexual women compared to heterosexual women, while gay and bisexual men had a higher prevalence of non-CM skin cancers compared to heterosexual men [27]. While our previous study in Atlantic Canada did not show any differences in self-reported skin cancer rates between LGBTQ+ and non-LGBTQ+ individuals [9], Singer et al. demonstrated a higher lifetime risk of any form of skin cancer among sexual minority men [28], reinforcing the importance of targeting this subgroup of the Canadian population through directed education and public health initiatives given our study’s demonstration of a greater burden of skin cancer risk factors amongst members of this population.

Only approximately half of our respondents reported using sunscreen often or always, and roughly one-third did not know whether their sunscreens had broad-spectrum coverage. Sunscreen use was notably lower among LGBTQ+ men compared to women, contrasting findings from the Lebanese LGBTQ+ population, where no difference was noted between gay and lesbian individuals [29]. Although sunscreen use is a key component of a comprehensive sun protection strategy, it should not be relied upon as the sole method of protection. Our research group has previously elucidated a paradox (i.e., the “sunscreen paradox”) whereby increased sunscreen usage was associated with increased sun exposure, conferring a false sense of protection and resulting in more sunburns and skin cancers [30]. Thus, public health initiatives and education efforts should focus on improving overall understanding of the limitations of sunscreen and reinforcing the importance of proper application, reapplication, and the necessity of complete sun blockade through sun-protective clothing, hats, sunglasses, and shaded structures in addition to sun avoidance during peak UV hours. The uncertainty regarding whether participants’ sunscreen provided broad-spectrum coverage (i.e., UVA and UVB coverage) was also highlighted in our previous study conducted in Manitoba [21]. Since both UVA and UVB radiation can contribute to the development of skin cancer, action should also be taken at the level of sunscreen manufacturers, who should strive to provide clear, comprehensive labeling for their products to facilitate consumer understanding. The American Academy of Dermatology (AAD) provides a comprehensive step-by-step guide on decoding sunscreen labels [31], while the Canadian Dermatology Association’s (CDA) Product Recognition Program [32] assists patients in selecting and understanding their sunscreens better. However, advocacy efforts at the national level remain limited with regard to the regulation of sunscreen products [33].

A considerable number of study respondents expressed concern about sunscreen polluting the ocean or containing toxic ingredients, which could deter sexual minorities from using sunscreen (Supplementary Table S2). Previous research has reported the presence of UV filters, particularly organic filters, in water sources, potentially posing a risk for coral reef bleaching or demise due to bioaccumulation and biomagnification [34,35]. However, organic UV filters have not been reported to cause clinically identifiable toxic effects on humans [36]. Given these findings, LGBTQ+ patients and members of the public should be encouraged to use inorganic UV filters while reinforcing the importance of sun-protective clothing and shade-seeking as non-chemical methods of sun protection. Furthermore, inorganic filters such as zinc oxide and titanium dioxide provide coverage against UVA, UVB, and visible light, conferring protection against both skin UV burns and skin aging [37,38].

Lifetime sunburns, blistering sunburns, tanning bed use, and recreational sun exposure were identified as common skin cancer risk factors among our respondents, emphasizing the important need for further advocacy to reduce UV exposure within the LGBTQ+ community. Some respondents believed that having a baseline tan protects against UV skin damage, while approximately half agreed they looked healthier or better with a tan. These sentiments are echoed by prior research, which also demonstrated that sexual minority men perceived tanning as sun-protective [39]. Although our earlier study [9] did not find differences between LGBTQ+ and non-LGBTQ+ individuals concerning tanning bed use, several studies have reported increased indoor tanning among sexual minority men compared to heterosexual men [11,12,28]. This discrepancy may be attributed to the study’s limited sample size, which focused only on Atlantic Canadians [9]. Among sexual minorities, motivations for indoor tanning included enhancing personal appearance or attractiveness, as well as mood elevation [28,39,40], while reasons for discontinuing tanning included concerns about skin aging, skin cancer development, and cost [40]. Therefore, discussing and emphasizing concerns about skin aging, the cost of skin rejuvenation treatments, and skin cancer risks associated with tanning could be valuable when counseling sexual minorities on the importance of sun protective practices.

In our study, the strongest correlations were those of sun exposure and sun protection habits with regard to the FSP. LGBTQ+ respondents with darker skin, classified as FSP IV–VI [25], exhibited significantly fewer sun protection habits and higher overall sun exposure compared to respondents with lighter skin tones (FSP I–III). This underscores the need to enhance public health efforts for sun safety among individuals with darker skin tones. Despite their relatively lower risk, UV radiation exposure remains a skin cancer risk factor for individuals with darker skin tones, in addition to exacerbating pigmentary skin disorders common in this demographic, such as melasma or post-inflammatory hyperpigmentation [41]. Given a general lack of awareness on the part of both patients and providers and higher difficulty in detecting skin cancers, African American patients are four times more likely to be diagnosed with advanced-stage melanoma, reinforcing the importance of disease prevention [42]. Furthermore, patients with darker skin tones are less likely to practice adequate photoprotection [43,44,45] due to reasons such as insufficient public education [43] and lack of counseling from physicians, including dermatologists, about the risks [43,44], limited representation of darker skin tones in medical education [45], and dissatisfaction with the white residue left by several sunscreens on the skin [43].

We also observed that respondents with lower educational rates and annual household incomes below CAD 50,000 had greater occupational sun exposure. In contrast, a higher level of education was associated with increased sunscreen use, and higher income was linked to more lifetime sunburns and tanning bed use. The latter finding aligns with a recent Canadian systematic review, which identified a correlation between higher socioeconomic status and increased CM incidence from 1979 to 2012 [46]. Similar results were also noted among LGBTQ+ individuals in Lebanon, where the likelihood of using sunscreen rose with higher levels of education [29]. Nonetheless, public education efforts focusing on sun safety should target populations across various education and income levels. Importantly, a previous study demonstrated that LGBTQ+ patients who received sun protection information from dermatologists were significantly more likely to use sunscreen compared to those receiving information from other sources [29]. Therefore, dermatologists should strive to establish trust with their LGBTQ+ patients and deliver culturally sensitive and well-informed guidance on skin and sun safety, including regular sunscreen use, emphasizing the importance of sun protective clothing, promoting sun avoidance/shade seeking, and reducing tanning bed use.

This study had limitations. Although our study includes a statistically representative number of LGBTQ+ respondents, some groups used for comparison analyses did not have enough respondents to make statistically significant comparisons. When using a two-sided test with approximately a 10% difference between proportions, an alpha of 0.05, and a power of 0.80, 250 respondents per group are needed to detect significant comparisons between groups. However, these analyses and findings were still included in our study, which is a statistical limitation. The groups with less than 250 respondents included men, gender-diverse individuals, FSP IV–VI, respondents without university degrees, and those with an annual household income of <CAD 50,000. Our study design may have introduced recall bias, as participants relied on memory when completing the surveys. Additionally, our open recruitment strategies could have led to selection bias, potentially influencing the results. For example, our study had a notably high number of individuals with a university degree when compared to the general population, which may be secondary to more educated individuals being aware of and willing to participate in scientific surveys. We did not stratify the LGBTQ+ participants based on which sexuality or gender they specifically identified with, which may allow for further insight (for instance, gay vs. lesbian vs. transgender). Lastly, the study’s four-year recruitment period means the findings represent a broader timeframe rather than a specific point in time, as would be expected in a cross-sectional study.

## 5. Conclusions

In conclusion, there are several concerning trends regarding UV exposure and sun protection within the LGBTQ+ community in Canada: ~60% report >10 lifetime sunburns and >1 blistering sunburn, ~35% had used a tanning bed at least once in their lifetime, and ~69% reported having a tan in the last 12 months. Concerningly, half of the participants thought that they looked better or healthier with a tan. Sunscreen was only worn regularly by approximately half of respondents, and a minority regularly sought shade or wore hats. These are significant risk factors for CM in the LGBTQ+ community, and they require urgent intervention through a multi-pronged approach involving healthcare providers, public health specialists, policymakers, and LGBTQ+ community leaders. In addition, important differences were highlighted based on gender, FPS, income, and education level. This study contributes important knowledge on ways in which LGBTQ+ individuals perceive skin cancer and sun protective behaviors, in addition to understanding how sexuality intersects with socioeconomic status in decision-making. These findings can be leveraged to better formulate focused public health campaigns with the goal of decreasing CM rates within the Canadian LGBTQ+ community.

## Figures and Tables

**Table 1:** Population demographic characteristics.

Variable	*N* (%)
Mean age (SD)	40.4 (17.1)
Median age (range)	38 (16–79)
Gender	
Men	248 (35.4)
Women	415 (59.3)
Gender diverse	37 (5.3)
Ethnicity *	
Non-Hispanic White or Euro-Canadian	593
Other	148
Annual household income	
<CAD 20,000	48 (6.9)
CAD 20,000–49,999	104 (14.9)
CAD 50,000–69,999	106 (15.1)
CAD 70,000–89,999	106 (15.1)
≥CAD 90,000	247 (35.3)
Highest level of education completed (n = 664)	
No high school	14 (2.1)
High school	126 (19.0)
CEGEP or college degree	109 (16.4)
University bachelor’s degree	252 (38.0)
Graduate or doctoral studies	157 (23.6)
Fitzpatrick skin phototype	
Type I	73 (10.4)
Type II	202 (28.9)
Type III	260 (37.1)
Type IV	133 (19.0)
Type V	29 (4.1)
Type VI	<10 (<1.4)
Province	
Newfoundland and Labrador	32 (4.6)
Prince Edward Island	<10 (<1.4)
Nova Scotia	121 (17.3)
New Brunswick	73 (10.4)
Quebec	140 (20.0)
Ontario	62 (8.9)
Manitoba	139 (19.9)
Saskatchewan	<10 (<1.4)
Alberta	<10 (<1.4)
British Colombia	118 (16.9)
Territories	0 (0.0)

Individuals who answered ‘I do not know’ or ‘I would rather not say’ were not included in the table. In total, 700 participants completed the survey. * Participants had the option to select more than one option. Other ethnicities include participants that identified as Black, Afro-Caribbean or African Canadian, Latino or Hispanic Canadian, East Asian or Asian Canadian, South Asian or Indian Canadian, Middle Eastern or Arab Canadian, Indigenous, or another ethnicity not otherwise specified. Only 664 participants responded to the question pertaining to education level, given that the survey had already been disseminated when the question was introduced.

## Data Availability

All available data is presented in the text of the manuscript and in Supplementary Materials.

## Supplementary Materials

The following supporting information can be downloaded at https://www.mdpi.com/article/10.3390/curroncol31120593/s1, Supplementary Tables S1–S7. **Table S1:** Ultraviolet exposure, melanoma risk factors, sun protection habits and level of worry for melanoma. **Table S2:** Participants reactions to various quotes. **Table S3:** Comparison of sun exposure, melanoma risk factors, sun protection habits and level of worry for melanoma between women vs. men vs. gender diverse respondents. **Table S4:** Comparison of sun exposure, melanoma risk factors, sun protection habits and level of worry for melanoma between individuals with a Fitzpatrick skin phototypes I-III vs. phototypes IV-VI. **Table S5:** Comparison of sun exposure, melanoma risk factors, sun protection habits and level of worry for melanoma between those that have completed a university degree vs. those that have not completed a university degree. **Table S6:** Comparison of sun exposure, melanoma risk factors, sun protection habits and level of worry for melanoma between individuals with an annual income ≥ CAD$50,000 vs. individuals with an annual income < CAD$50,000. **Table S7:** Comparison of UV exposure, melanoma risk factors, sun protection habits, and melanoma-related worry between participants in the current study and those from two previous studies conducted by our research group in the Canadian Atlantic provinces and Manitoba.
