# Supplementary material for: Investigating Skin Cancer Risk and Sun Safety Practices Among LGBTQ+ Communities in Canada
Source: Curr Oncol. 2024 Dec 19;31(12):8039–53. doi: 10.3390/curroncol31120593 (PMC11674060; doi:10.3390/curroncol31120593)
Supplement: Supplementary file 1 [file curroncol-31-00593-s001.zip › curroncol-3266514-supplementary.pdf]

Supplementary Table S1. Ultraviolet exposure, melanoma risk factors, sun protection habits and level of worry for melanoma. Individuals that answered 'I do not know' or 'I would rather not say' were not included in the table. In total, 700 participants completed the survey.

Variable	N (%)
Personal history of skin cancer	40 (5.7)
Cutaneous Melanoma	14 (35.0)
Squamous cell carcinoma (SCC)	<10 (<1.4)
Basal cell carcinoma (BCC)	17 (42.5)
Family history of skin cancer	159 (22.7)
Lifetime sunburns (more than 10)	420 (60.0)
Lifetime blistering sunburns (1 or more)	418 (58.2)
Tanning bed use (1 or more)	240 (34.3)
Sun exposure ('high' or 'very high')	
Total	125 (17.9)
Recreational	186 (26.6)
Occupational	43 (6.1)
Tan in the last 12 months	482 (68.9)
Spends time in the sun daily or multiple days per week to get a tan or to feel good (excluding vacation)	76 (10.9)
Spends time in the sun daily or multiple days per week to get a tan or to feel good (vacation)	206 (29.5)
Sun protection ('often' or 'always')	
Sunscreen	356 (50.9)
Long sleeves	452 (64.6)
Hats	177 (25.3)
Shade	227 (32.4)
Sunglasses	389 (55.6)
Sunscreen characteristics	
Broad spectrum (UVA and UVB)	444 (63.4)
SPF ≥ 30	604 (86.3)
Skin check	516 (73.7)
Reaction to a new mole	
Family doctor visit	195 (27.9)
Check by friend/family member	327 (46.7)
Ignore	62 (8.9)
Search the internet	108 (15.4)
Worry if mole	
Is irregular in shape	670 (95.7)
Changes colour	681 (97.3)
Grows in size	674 (96.3)

Supplementary Table S2. Participants reactions to various quotes. Individuals that answered 'I do not know' or 'I would rather not say' were not included in the table. In total, 700 participants completed the survey.

Quote	N (%)
"Having a base tan is protective against the sun's UV radiation/skin damage"	
Agree and strongly agree	111 (15.9)
Disagree and strongly disagree	432 (61.7)
"It is rare to get melanoma before the age of 35"	

Agree and strongly agree	104 (14.9)
Disagree and strongly disagree	379 (54.1)
"I check my skin on a regular basis for abnormal moles"	
Agree and strongly agree	406 (58.0)
Disagree and strongly disagree	196 (28.0)
"Sunscreens pollute the oceans"	
Agree and strongly agree	242 (34.6)
Disagree and strongly disagree	180 (25.7)
"Sunscreens contain toxic ingredients"	
Agree and strongly agree	150 (21.4)
Disagree and strongly disagree	312 (44.6)
"I look better and/or healthier with a tan"	
Agree and strongly agree	350 (50.0)
Disagree and strongly disagree	197 (28.1)
"Tanning beds are a safer, more controlled way to get a tan than from the sun"	
Agree and strongly agree	25 (3.6)
Disagree and strongly disagree	579 (82.7)

Supplementary Table S3. Comparison of sun exposure, melanoma risk factors, sun protection habits and level of worry for melanoma between women (n=415) vs men (n=248) vs gender diverse respondents (n=37). Individuals that answered 'I do not know' or 'I would rather not say' were not included in the analysis. The odds ratios are adjusted (aOR) for age. Comparisons that reached statistical significance are marked with an asterisk (*).

Variable	Women N (%)	Men N (%)	Gender Diverse N (%)	aOR[a] (95% CI)	p-value[a]	aOR[b] (95% CI)	p-value[b]	aOR[c] (95% CI)	p-value[c]
Personal history of skin cancer	17 (4.1)	23 (9.3)	0 (0.0)	0.43 (0.22-0.86)	0.02*	-	-	-	-
Family history of skin cancer	101 (24.3)	49 (19.8)	<10 (<27.0)	1.33 (0.90-1.98)	0.15	1.33 (0.59-3.01)	0.50	1.59 (0.68-3.71)	0.28
Lifetime sunburns (more than 10)	247 (59.5)	149 (60.1)	24 (64.9)	1.06 (0.76-1.47)	0.75	1.39 (0.67-2.89)	0.37	1.58 (0.74-3.38)	0.24
Lifetime blistering sunburns (1 or more)	271 (65.3)	122 (49.2)	25 (67.6)	2.03 (1.45-2.85)	<0.001*	1.37 (0.66-2.87)	0.40	2.31 (1.09-4.90)	0.03*
Tanning bed use (1 or more)	148 (35.7)	90 (36.3)	<10 (<27.0)	1.03 (0.73-1.44)	0.88	0.13 (0.03-0.54)	0.005*	0.13 (0.03-0.54)	0.005*
Sun exposure ('high' or 'very high')									
Total	68 (16.4)	52 (21.0)	<10 (<27.0)	0.75 (0.50-1.12)	0.15	0.85 (0.32-2.27)	0.74	0.68 (0.25-1.86)	0.45

Recreational	114 (27.5)	64 (25.8)	<10 (<27.0)	1.06 (0.74-1.51)	0.76	0.64 (0.28-1.46)	0.29	0.76 (0.32-1.77)	0.52
Occupational	22 (5.3)	21 (8.5)	0 (0.0)	0.60 (0.32-1.12)	0.11	-	-	-	-
Tan in the last 12 months	282 (68.0)	177 (71.4)	23 (62.2)	0.85 (0.60-1.21)	0.37	0.70 (0.35-1.41)	0.32	0.64 (0.31-1.34)	0.24
Spends time in the sun daily or multiple days per week to get a tan or to feel good (excluding vacation)	42 (10.1)	32 (12.9)	<10 (<27.0)	0.77 (0.47-1.26)	0.30	0.53 (0.12-2.29)	0.39	0.45 (0.10-2.01)	0.30
Spends time in the sun daily or multiple days per week to get a tan or to feel good (vacation)	115 (27.7)	87 (35.1)	<10 (<27.0)	0.73 (0.51-1.03)	0.07	0.31 (0.11-0.92)	0.04*	0.22 (0.07-0.67)	0.007*
Sun protection ('often' or 'always')									
Sunscreen	229 (55.2)	103 (41.5)	24 (64.9)	1.70 (1.22-2.35)	0.001*	1.21 (0.59-2.47)	0.61	2.08 (0.99-4.36)	0.052
Long sleeves	237 (57.1)	191 (77.0)	24 (64.9)	0.40 (0.28-0.57)	<0.001*	1.47 (0.72-2.97)	0.29	0.58 (0.27-1.23)	0.15
Hats	99 (23.9)	70 (28.2)	<10 (<27.0)	0.84 (0.58-1.22)	0.35	1.25 (0.54-2.91)	0.60	1.08 (0.45-2.58)	0.87
Shade	140 (33.7)	70 (28.2)	17 (45.9)	1.35 (0.95-1.91)	0.09	1.86 (0.93-3.69)	0.08	2.97 (1.41-6.24)	0.004*
Sunglasses	221 (53.3)	148 (59.7)	20 (54.1)	0.79 (0.57-1.08)	0.14	1.14 (0.58-2.26)	0.70	0.89 (0.44-1.81)	0.75
Sunscreen used is SPF ≥ 30	374 (90.1)	199 (80.2)	31 (83.8)	2.10 (1.33-3.33)	0.002*	0.39 (0.15-1.05)	0.06	0.98 (0.38-2.54)	0.96
Skin check	313 (75.4)	176 (71.0)	27 (73.0)	1.32 (0.93-1.89)	0.12	1.08 (0.49-2.38)	0.86	1.37 (0.61-3.09)	0.45
Worry if mole									
Is irregular in shape	399 (96.1)	234 (94.4)	37 (100.0)	1.89 (0.69-5.16)	0.21	-	-	-	-
Changes colour	404 (97.3)	240 (96.8)	37 (100.0)	1.42 (0.38-5.37)	0.60	-	-	-	-

Grows in size	398 (95.9)	239 (96.4)	37 (100.0)	0.80 (0.27-2.35)	0.69	-	-	-	-

a: Comparing women to men.
b: Comparing gender diverse respondents to women.
c: Comparing gender diverse respondents to men.

Supplementary Table S4. Comparison of sun exposure, melanoma risk factors, sun protection habits and level of worry for melanoma between individuals with a Fitzpatrick skin phototypes I-III (n=535) vs. phototypes IV-VI (n=164). Individuals that answered 'I do not know' or 'I would rather not say' were not included in the analysis. The odds ratios (OR) are adjusted for age and gender. Comparisons that reached statistical significance are marked with an asterisk (*).

Variable	Fitzpatrick I-III N (%)	Fitzpatrick IV-VI N (%)	Adjusted OR (95% CI)	p-value
Personal history of skin cancer	35 (6.5)	<10 (<6.1)	2.21 (0.81-6.06)	0.12
Family history of skin cancer	131 (24.5)	28 (17.1)	1.57 (0.97-2.45)	0.06
Lifetime sunburns (more than 10)	373 (69.7)	47 (28.7)	6.25 (4.20-9.30)	<0.001*
Lifetime blistering sunburns (1 or more)	349 (65.2)	69 (42.1)	2.51 (1.72-3.67)	<0.001*
Tanning bed use (1 or more)	188 (35.1)	52 (31.7)	1.07 (0.72-1.58)	0.74
Sun exposure ('high' or 'very high')				
Total	87 (16.3)	38 (23.2)	0.64 (0.41-0.98)	0.04*
Recreational	131 (24.5)	54 (32.9)	0.68 (0.46-1.00)	0.051
Occupational	30 (5.6)	13 (7.9)	0.70 (0.36-1.39)	0.31
Tan in the last 12 months	361 (67.5)	121 (73.8)	0.67 (0.45-1.02)	0.06
Spends time in the sun daily or multiple days per week to get a tan or to feel good (excluding vacation)	60 (11.2)	16 (9.8)	1.15 (0.64-2.07)	0.64
Spends time in the sun daily or multiple days per week to get a tan or to feel good (vacation)	157 (29.4)	49 (29.9)	1.01 (0.68-1.50)	0.97
Sun protection ('often' or 'always')				
Sunscreen	290 (54.2)	65 (39.6)	2.00 (1.37-2.90)	<0.001*
Long sleeves	355 (66.4)	96 (58.5)	1.46 (1.01-2.11)	0.045*
Hats	147 (27.5)	29 (17.7)	1.72 (1.08-2.74)	0.02*

Shade	187 (35.0)	40 (24.4)	1.63 (1.08-2.44)	0.02*
Sunglasses	309 (57.8)	79 (48.2)	1.46 (1.02-2.07)	0.04*
Sunscreen used is SPF ≥ 30	477 (89.2)	126 (76.8)	2.65 (1.65-4.27)	<0.001*
Skin check	397 (74.2)	118 (72.0)	1.10 (0.74-1.63)	0.65
Worry if mole				
Is irregular in shape	511 (95.5)	158 (96.3)	0.61 (0.17-2.21)	0.45
Changes colour	519 (97.0)	161 (98.2)	0.82 (0.17-4.03)	0.81
Grows in size	515 (96.3)	158 (96.3)	0.69 (0.19-2.46)	0.56

Supplementary Table S5. Comparison of sun exposure, melanoma risk factors, sun protection habits and level of worry for melanoma between those that have completed a university degree (n=409) vs. those that have not completed a university degree (n=249). Individuals that answered 'I do not know' or 'I would rather not say' were not included in the analysis. The odds ratios (OR) are adjusted for age and gender. Comparisons that reached statistical significance are marked with an asterisk (*).

Variable	University N (%)	No university N (%)	Adjusted OR (95% CI)	p-value
Personal history of skin cancer	23 (5.6)	13 (5.2)	1.16 (0.54-2.47)	0.70
Family history of skin cancer	100 (24.4)	50 (20.1)	1.22 (0.82-1.80)	0.33
Lifetime sunburns (more than 10)	256 (62.6)	140 (56.2)	1.39 (1.00-1.94)	0.053
Lifetime blistering sunburns (1 or more)	250 (61.1)	142 (57.0)	1.15 (0.82-1.62)	0.42
Tanning bed use (1 or more)	147 (35.9)	75 (30.1)	1.21 (0.85-1.72)	0.30
Sun exposure ('high' or 'very high')				
Total	69 (16.9)	47 (18.9)	0.84 (0.56-1.27)	0.41
Recreational	104 (25.4)	71 (28.5)	0.90 (0.63-1.29)	0.56
Occupational	17 (4.2)	22 (8.8)	0.42 (0.22-0.81)	0.01*
Tan in the last 12 months	280 (68.5)	171 (68.7)	0.99 (0.70-1.41)	0.97
Spends time in the sun daily or multiple days per week to get a tan or to feel good (excluding vacation)	34 (8.3)	34 (13.7)	0.55 (0.33-0.91)	0.02*
Spends time in the sun daily or multiple days per week to get a tan or to feel good (vacation)	120 (29.3)	71 (28.6)	0.92 (0.64-1.33)	0.49

Sun protection ('often' or 'always')				
Sunscreen	222 (54.3)	107 (43.0)	1.89 (1.35-2.65)	0.002*
Long sleeves	270 (66.0)	152 (61.0)	1.17 (0.84-1.64)	0.36
Hats	105 (25.7)	60 (24.1)	1.03 (0.70-1.51)	0.89
Shade	124 (30.3)	82 (32.9)	0.87 (0.62-1.23)	0.44
Sunglasses	223 (54.5)	138 (55.4)	0.92 (0.66-1.26)	0.59
Sunscreen used is SPF ≥ 30	258 (87.5)	214 (85.9)	1.21 (0.75-1.95)	0.44
Skin check	309 (75.6)	176 (70.7)	1.23 (0.85-1.76)	0.27
Worry if mole				
Is irregular in shape	392 (95.8)	237 (95.2)	2.58 (0.90-7.42)	0.08
Changes colour	398 (97.3)	243 (97.6)	0.92 (0.21-3.98)	0.91
Grows in size	395 (96.6)	237 (95.2)	0.89 (0.31-2.54)	0.82

Supplementary Table S6. Comparison of sun exposure, melanoma risk factors, sun protection habits and level of worry for melanoma between individuals with an annual income ≥ CAD$50,000 (n=459) vs. individuals with an annual income < CAD$50,000 (n=152). Individuals that answered 'I do not know' or 'I would rather not say' were not included in the analysis. The odds ratios (OR) are adjusted for age and gender. Comparisons that reached statistical significance are marked with an asterisk (*).

Variable	Income ≥$50,000 N (%)	Income <$50,000 N (%)	Adjusted OR (95% CI)	p-value
Personal history of skin cancer	27 (5.9)	10 (6.6)	0.78 (0.33-1.80)	0.55
Family history of skin cancer	105 (22.9)	37 (24.3)	0.81 (0.52-1.27)	0.36
Lifetime sunburns (more than 10)	293 (63.8)	80 (52.6)	1.61 (1.10-2.38)	0.02*
Lifetime blistering sunburns (1 or more)	279 (60.8)	92 (60.5)	0.95 (0.63-1.42)	0.79
Tanning bed use (1 or more)	185 (40.3)	34 (22.4)	2.03 (1.30-3.17)	0.002*
Sun exposure ('high' or 'very high')				
Total	83 (18.1)	24 (15.8)	1.10 (0.67-1.83)	0.70
Recreational	120 (26.1)	36 (23.7)	1.25 (0.81-1.94)	0.32

Occupational	23 (5.0)	16 (10.5)	0.40 (0.20-0.78)	0.008*
Tan in the last 12 months	323 (70.4)	101 (66.4)	1.19 (0.79-1.78)	0.41
Spends time in the sun daily or multiple days per week to get a tan or to feel good (excluding vacation)	49 (10.7)	14 (9.2)	1.08 (0.57-2.03)	0.82
Spends time in the sun daily or multiple days per week to get a tan or to feel good (vacation)	148 (32.2)	36 (23.7)	1.48 (0.95-2.30)	0.08
Sun protection ('often' or 'always')				
Sunscreen	242 (52.7)	77 (50.7)	1.39 (0.94-2.06)	0.10
Long sleeves	303 (66.0)	89 (58.6)	1.25 (0.84-1.84)	0.27
Hats	119 (25.9)	33 (21.7)	1.07 (0.67-1.71)	0.78
Shade	141 (30.7)	51 (33.6)	0.85 (0.57-1.27)	0.43
Sunglasses	277 (60.3)	66 (43.4)	1.86 (1.28-2.72)	0.001*
Sunscreen used is SPF ≥ 30	405 (88.2)	127 (83.6)	1.71 (0.99-2.95)	0.054
Skin check	350 (76.3)	109 (71.7)	1.24 (0.81-1.90)	0.31
Worry if mole				
Is irregular in shape	447 (97.4)	141 (92.8)	3.52 (1.12-11.07)	0.03*
Changes colour	448 (97.6)	146 (96.1)	1.40 (0.33-5.91)	0.65
Grows in size	442 (96.3)	149 (98.0)	0.68 (0.18-2.54)	0.57

Supplementary Table S7. Comparison of UV exposure, melanoma risk factors, sun protection habits, and melanoma-related worry between participants in the current study and those from two previous studies conducted by our research group in the Canadian Atlantic provinces and Manitoba.

Variable<br>n (%)	Current study findings	Lagacé et al.[9]	Lagacé et al.[21]
Studied population	Canadian LGBTQ+	Atlantic provinces	Manitoba
Total sample size	700	7,861	3,347
Number of LGBTQ+ respondents	700 (100)	226 (2.9)	139 (4.2)
Personal history of skin cancer	40 (5.7)	1,036 (13.2)	285 (8.5)
Family history of skin cancer	159 (22.7)	2,548 (32.4)	943 (28.2)
Lifetime sunburns (more than 10)	420 (60.0)	5,154 (65.6)	2,267 (67.7)
Lifetime blistering sunburns (1 or more)	418 (58.2)	5,572 (70.9)	2,250 (67.2)
Tanning bed use (1 or more)	240 (34.3)	3,717 (47.3)	1,741 (52.0)

Sun exposure ('high' or 'very high')			
Total	125 (17.9)	1,612 (20.5)	682 (20.4)
Recreational	186 (26.6)	1,777 (22.6)	788 (23.6)
Occupational	43 (6.1)	278 (3.5)	128 (3.8)
Tan in the last 12 months	482 (68.9)	5,819 (74.0)	2,641 (78.9)
Spends time in the sun daily or multiple days per week to get a tan or to feel good (excluding vacation)	76 (10.9)	-	453 (13.5)
Spends time in the sun daily or multiple days per week to get a tan or to feel good (vacation)	206 (29.5)	-	1,061 (31.8)
Sun protection ('often' or 'always')			
Sunscreen	356 (50.9)	3,650 (46.4)	1,555 (46.5)
Long sleeves	452 (64.6)	4,903 (62.2)	1,932 (57.7)
Hats	177 (25.3)	2,694 (34.3)	1,038 (31.0)
Shade	227 (32.4)	2,981 (37.9)	1,131 (33.8)
Sunglasses	389 (55.6)	5,582 (71.0)	2,361 (70.5)
Sunscreen characteristics			
Broad spectrum	444 (63.4)	5,229 (66.5)	2,084 (62.3)
SPF ≥ 30	604 (86.3)	6,251 (79.5)	2,714 (81.1)
Skin check	516 (73.7)	6,563 (83.5)	2,775 (82.9)
Reaction to a new mole			
Family doctor visit	195 (27.9)	3,098 (39.4)	1,278 (38.2)
Check by friend/family member	327 (46.7)	3,657 (46.5)	1,472 (44.0)
Ignore	62 (8.9)	335 (4.3)	186 (5.6)
Search the internet	108 (15.4)	-	305 (9.1)
Worry if mole			
Is irregular in shape	670 (95.7)	7,569 (96.3)	3,206 (95.8)
Changes colour	681 (97.3)	7,674 (97.6)	3,246 (97.0)
Grows in size	674 (96.3)	7,675 (97.6)	3,270 (97.7)

Supplementary Table References:

9. Lagacé F, Noorah BN, Conte S, et al. Assessing Skin Cancer Risk Factors, Sun Safety Behaviors and Melanoma Concern in Atlantic Canada: A Comprehensive Survey Study. *Cancers (Basel)*. Jul 25 2023;15(15)doi:10.3390/cancers15153753

21. Lagacé F, Conte S, Mija LA, et al. A Comprehensive Analysis of Skin Cancer Concerns and Protective Practices in Manitoba, Canada, Highlights Lack of Skin Cancer Awareness and Predominance of High-Risk Sun Exposure Behaviors. *Cancers (Basel)*. Sep 5 2024;16(17)doi:10.3390/cancers16173093
